# The genome sequence of field madder, *Sherardia arvensis *L., 1753 (Rubiaceae)

**DOI:** 10.12688/wellcomeopenres.21027.1

**Published:** 2024-03-01

**Authors:** Maarten J. M. Christenhusz, Royal Botanic Gardens Kew Genome Acquisition Lab, Plant Genome Sizing collective, Darwin Tree of Life Barcoding collective, Wellcome Sanger Institute Tree of Life Management, Samples and Laboratory team, Wellcome Sanger Institute Scientific Operations: Sequencing Operations, Wellcome Sanger Institute Tree of Life Core Informatics team, Tree of Life Core Informatics collective, Darwin Tree of Life Consortium

**Affiliations:** 1 Royal Botanic Gardens Kew, Richmond, England, UK; 2 Curtin University, Perth, Western Australia, Australia

**Keywords:** Sherardia arvensis, field madder, genome sequence, chromosomal, Gentianales

## Abstract

We present a genome assembly from an individual *Sherardia arvensis* (field madder; Tracheophyta; Magnoliopsida; Gentianales; Rubiaceae). The genome sequence is 440.9 megabases in span. Most of the assembly is scaffolded into 11 chromosomal pseudomolecules. The mitochondrial and plastid genome assemblies have lengths of 203.98 kilobases and 152.73 kilobases in length, respectively.

## Species taxonomy

Eukaryota; Viridiplantae; Streptophyta; Streptophytina; Embryophyta; Tracheophyta; Euphyllophyta; Spermatophyta; Magnoliopsida; Mesangiospermae; eudicotyledons; Gunneridae; Pentapetalae; asterids; lamiids; Gentianales; Rubiaceae; Rubioideae; Rubieae; *Sherardia*; *Sherardia arvensis* L., 1753 (NCBI:txid29803).

## Background

Easily overlooked, the field madder, *Sherardia arvensis* is a small, annual plant found in open, dry grassland, sheltered cliffs, sand dunes, arable fields, waste ground, waysides and verges. It is the only species in the genus *Sherardia*, which is distinguished from other Rubiaceae by the fused involucral bracts forming flower heads. The genus was named in honour of English botanist and consul William Sherard (1659–1728). *Sherardia arvensis* was formerly frequent, but is now much decreased in Britain and Ireland due to agricultural intensification since the 1950s ( OABIF, 2022; Sutcliffe & Kay, 2000). Studies of the genetic variation within and between populations from different habitats found that a clear correlation between geographical and genetic distances could was only found for the indigenous populations studied and was absent in sites characterised by more intensive agricultural practices ( Listl & Reisch, 2014).

*Sherardia arvensis* makes a mat of trailing, quadrangular stems up to 40 cm long. The leaves, approximately 1 cm in length, are placed in whorls of four to six and are roughly hairy. Flowers are grouped with up to three together in leaf axils surrounded by involucral bracts. The tiny (3 mm wide) petals are lilac-blue and fused into a funnel with four pointed lobes. Flowers are pollinated by flies, but will also regularly self-pollinate. Fruits are composed of two indehiscent nutlets.

Field madder is found naturally throughout Europe and north Africa, and east into west and central Asia. It has been commonly introduced as an agricultural weed in other temperate regions of North and South America, East and South Africa, Bermuda, Taiwan, Hawaii, Australia, New Zealand and sub-Antarctic islands ( POWO, 2022). In Britain and Ireland, it is likely that it is native only in western coastal habitats, whereas elsewhere it is an archaeophyte ( OABIF, 2022).

The roots are reported to have been used by the ancient Celts as a source of a red or pink dye, but the quality is inferior to another species in the Rubiaceae - the dyer’s madder ( *Rubia tinctorum*; Moerman, 1998; Riley, 1997) which has been much more widely used ( Biertümpfel & Wurl, 2009).

We here present the first complete genome of *Sherardia arvensis*. This will aid in understanding of how the native populations have spread across Britain and beyond.

## Genome sequence report

**Figure 1.**
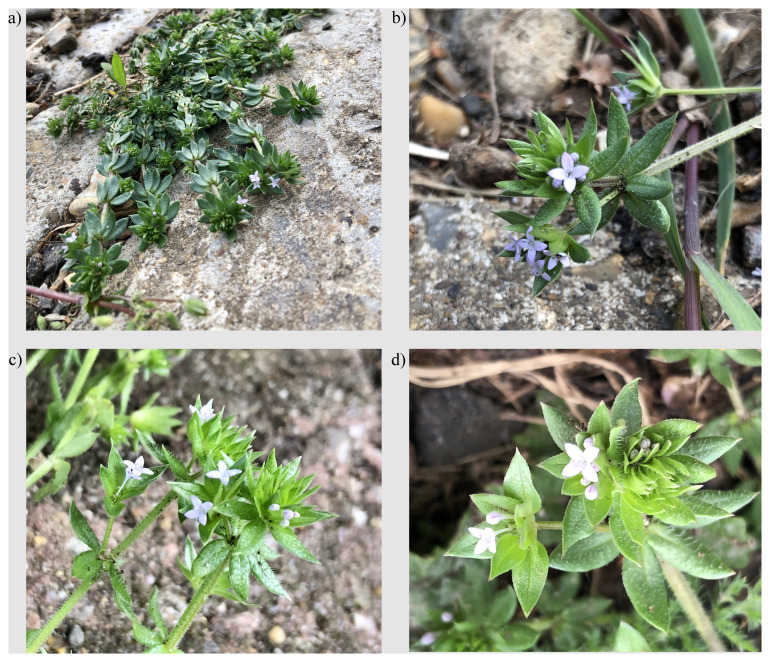
Photographs of the *Sherardia arvensis* (daSheArve1) specimen used for genome sequencing.

The genome was sequenced from a specimen of *Sherardia arvensis* ( Figure 1) collected from Teddington Lock, Surrey, UK (51.43, –0.33). Using flow cytometry, the genome size (1C-value) was estimated to be 0.60 pg, equivalent to 580 Mb. A total of 76-fold coverage in Pacific Biosciences single-molecule HiFi long reads was generated. Primary assembly contigs were scaffolded with chromosome conformation Hi-C data. Manual assembly curation corrected 4 missing joins or mis-joins.

**Figure 2.**
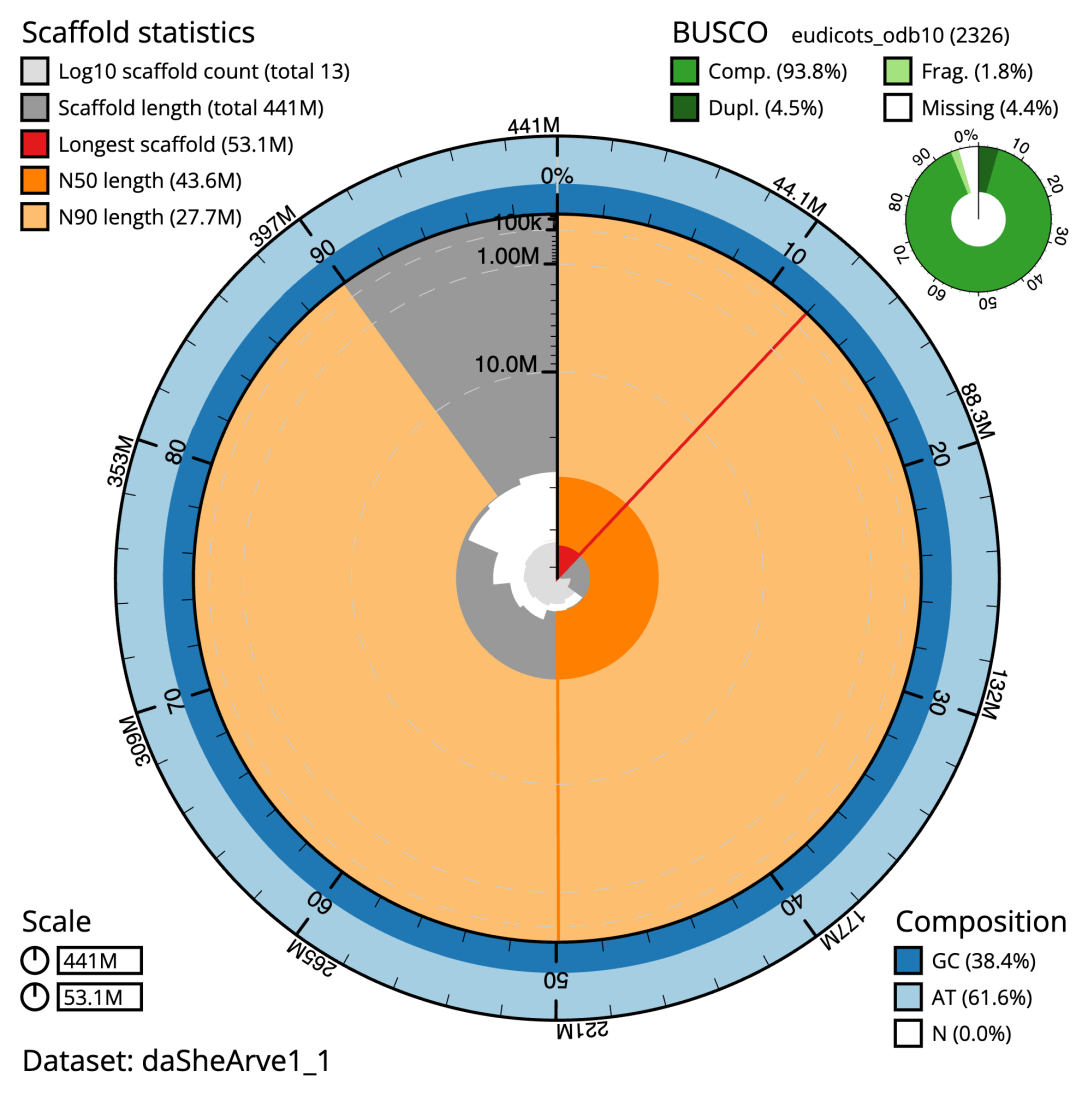
Genome assembly of *Sherardia arvensis*, daSheArve1.1: metrics. The BlobToolKit snail plot shows N50 metrics and BUSCO gene completeness. The main plot is divided into 1,000 size-ordered bins around the circumference with each bin representing 0.1% of the 441,296,019 bp assembly. The distribution of scaffold lengths is shown in dark grey with the plot radius scaled to the longest scaffold present in the assembly (53,145,315 bp, shown in red). Orange and pale-orange arcs show the N50 and N90 scaffold lengths (43,593,172 and 27,662,378 bp), respectively. The pale grey spiral shows the cumulative scaffold count on a log scale with white scale lines showing successive orders of magnitude. The blue and pale-blue area around the outside of the plot shows the distribution of GC, AT and N percentages in the same bins as the inner plot. A summary of complete, fragmented, duplicated and missing BUSCO genes in the eudicots_odb10 set is shown in the top right. An interactive version of this figure is available at https://blobtoolkit.genomehubs.org/view/daSheArve1_1/dataset/daSheArve1_1/snail.

**Figure 3.**
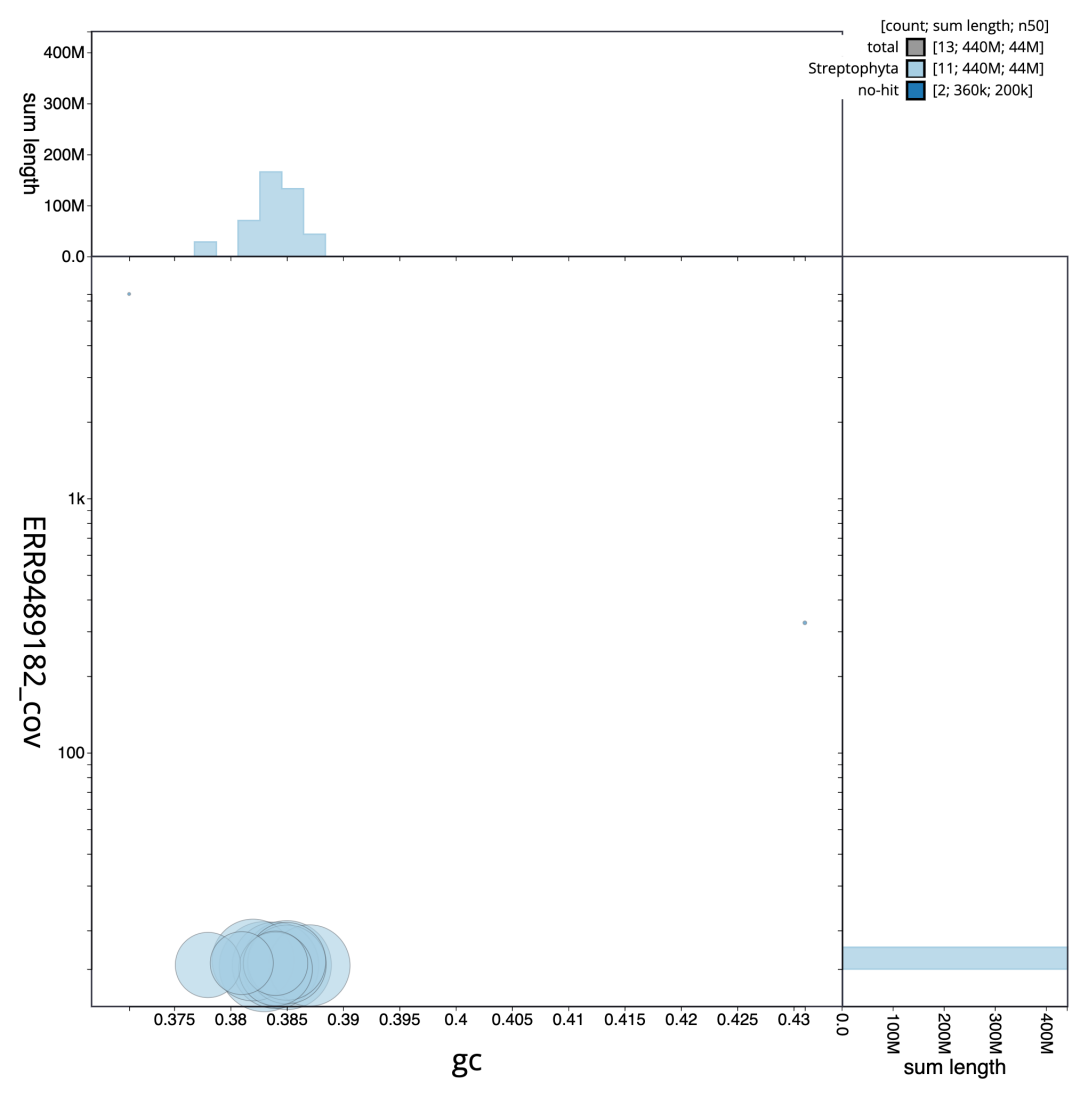
Genome assembly of *Sherardia arvensis*, daSheArve1.1: BlobToolKit GC-coverage plot. Scaffolds are coloured by phylum. Circles are sized in proportion to scaffold length. Histograms show the distribution of scaffold length sum along each axis. An interactive version of this figure is available at https://blobtoolkit.genomehubs.org/view/daSheArve1_1/dataset/daSheArve1_1/blob.

**Figure 4.**
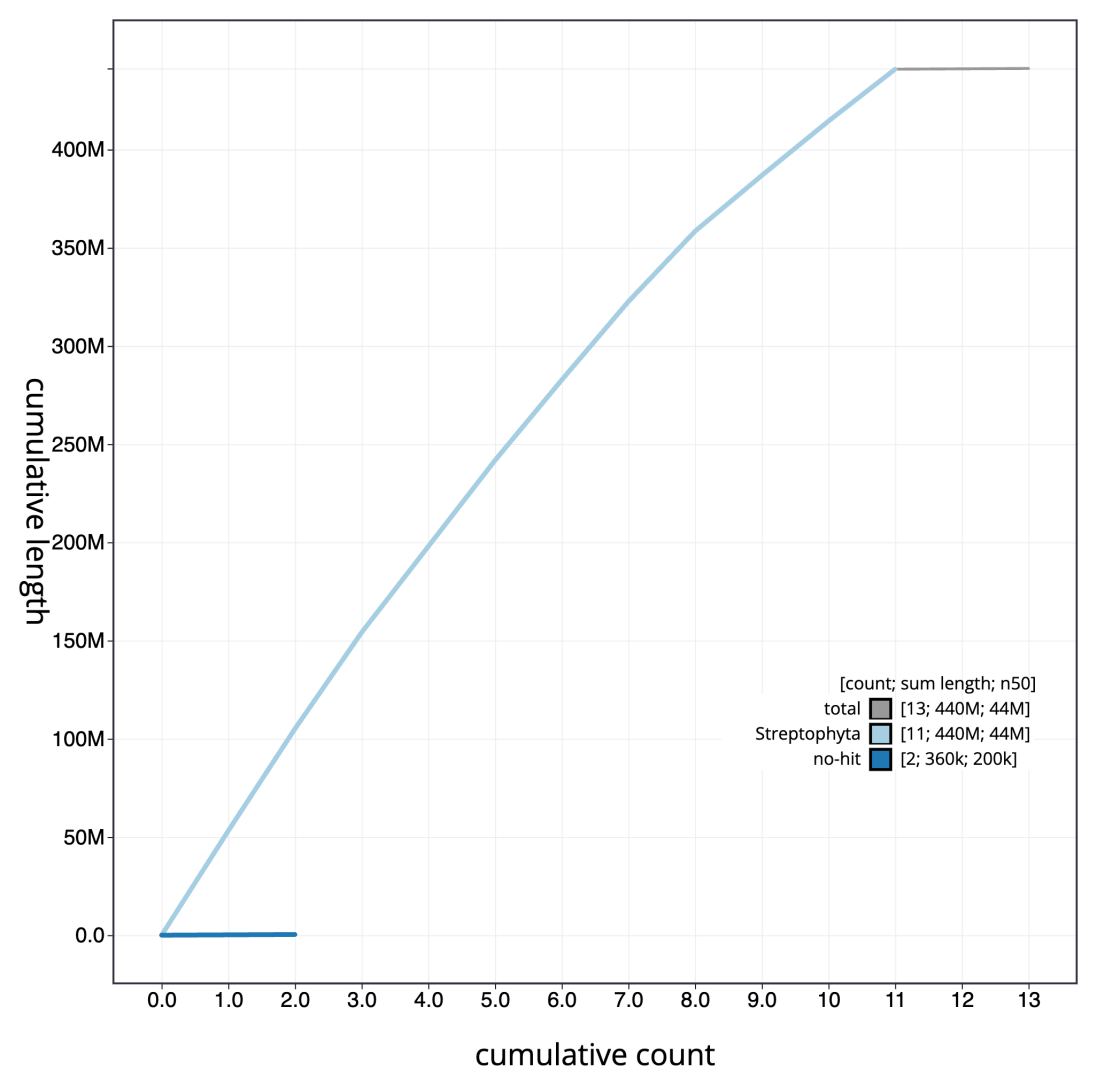
Genome assembly of *Sherardia arvensis*, daSheArve1.1: BlobToolKit cumulative sequence plot. The grey line shows cumulative length for all scaffolds. Coloured lines show cumulative lengths of scaffolds assigned to each phylum using the buscogenes taxrule. An interactive version of this figure is available at https://blobtoolkit.genomehubs.org/view/daSheArve1_1/dataset/daSheArve1_1/cumulative.

**Figure 5.**
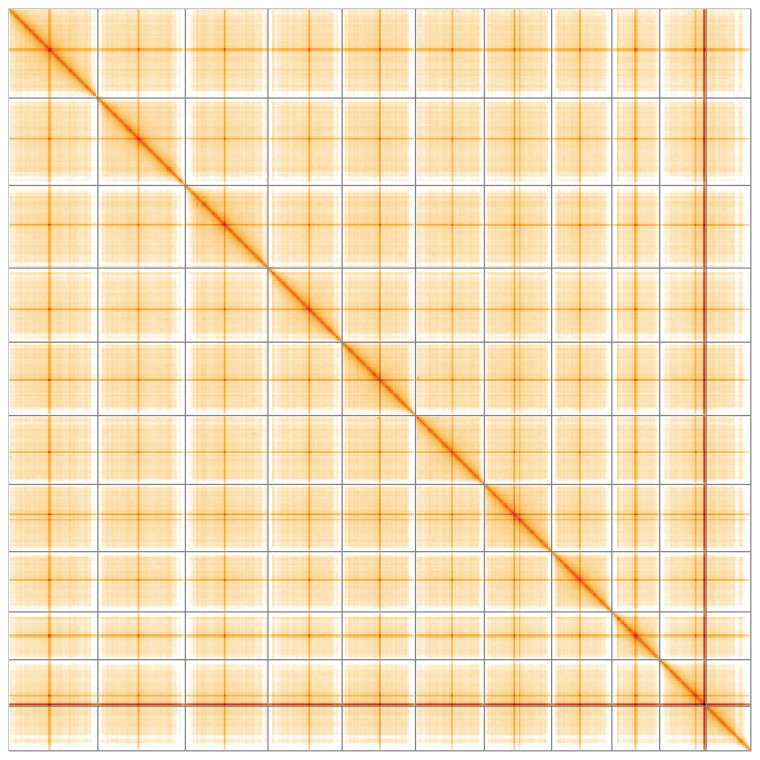
Genome assembly of *Sherardia arvensis*, daSheArve1.1: Hi-C contact map of the daSheArve1.1 assembly, visualised using HiGlass. Chromosomes are shown in order of size from left to right and top to bottom. An interactive version of this figure may be viewed at https://genome-note-higlass.tol.sanger.ac.uk/l/?d=Ny8eTdTwQGWpt06JQDWCLw.

The final assembly has a total length of 440.9 Mb in 11 sequence scaffolds with a scaffold N50 of 43.6 Mb ( Table 1). The snail plot in Figure 2 provides a summary of the assembly statistics, while the distribution of assembly scaffolds on GC proportion and coverage is shown in Figure 3. The cumulative assembly plot in Figure 4 shows curves for subsets of scaffolds assigned to different phyla. Most (99.91%) of the assembly sequence was assigned to 11 chromosomal-level scaffolds, which is consistent with the diploid chromosome count from British material reported for this species (i.e. 2 *n* = 22; Maude, 1939). Chromosome-scale scaffolds confirmed by the Hi-C data are named in order of size ( Figure 5; Table 2). While not fully phased, the assembly deposited is of one haplotype. Contigs corresponding to the second haplotype have also been deposited. The mitochondrial and plastid genomes were also assembled and can be found as contigs within the multifasta file of the genome submission.

**Table 1.** Genome data for *Sherardia arvensis*, daSheArve1.1.

Project accession data		
Assembly identifier	daSheArve1.1	
Species	*Sherardia arvensis*	
Specimen	daSheArve1	
NCBI taxonomy ID	29803	
BioProject	PRJEB50877	
BioSample ID	SAMEA9143091	
Isolate information	daSheArve1: leaves (DNA, Hi-C and RNA sequencing)	
Assembly metrics *		*Benchmark*
Consensus quality (QV)	71.9	≥ 50
*k*-mer completeness	100.0%	≥ 95%
BUSCO **	C:93.8%[S:89.3%,D:4.5%], F:1.8%,M:4.4%,n:2,326	C ≥ 95%
Percentage of assembly mapped to chromosomes	99.91%	≥ 95%
Sex chromosomes	None	*localised homologous pairs*
Organelles	Mitochondrial genome: 203.98 kb Plastid genome: 152.73 kb	*complete single alleles*
Raw data accessions		
PacificBiosciences SEQUEL II	ERR8705858, ERR9489182	
Hi-C Illumina	ERR8702785	
PolyA RNA-Seq Illumina	ERR10890681	
Genome assembly		
Assembly accession	GCA_948330725.1	
*Accession of alternate haplotype*	GCA_948329965.1	
Span (Mb)	440.9	
Number of contigs	35	
Contig N50 length (Mb)	18.9	
Number of scaffolds	11	
Scaffold N50 length (Mb)	43.6	
Longest scaffold (Mb)	53.15	

* Assembly metric benchmarks are adapted from column VGP-2020 of “Table 1: Proposed standards and metrics for defining genome assembly quality” from ( Rhie *et al.*, 2021). ** BUSCO scores based on the eudicots_odb10 BUSCO set using version 5.3.2. C = complete [S = single copy, D = duplicated], F = fragmented, M = missing, n = number of orthologues in comparison. A full set of BUSCO scores is available at https://blobtoolkit.genomehubs.org/view/daSheArve1_1/dataset/daSheArve1_1/busco.

**Table 2.** Chromosomal pseudomolecules in the genome assembly of *Sherardia arvensis*, daSheArve1.

INSDC accession	Chromosome	Length (Mb)	GC%
OX415235.1	1	53.15	38.5
OX415236.1	2	51.95	38.5
OX415237.1	3	49.06	38.5
OX415238.1	4	44.03	38.0
OX415239.1	5	43.59	38.5
OX415240.1	6	40.91	38.5
OX415241.1	7	39.93	38.5
OX415242.1	8	35.8	38.5
OX415244.1	9	27.66	38.5
OX415243.1	10	28.39	38.0
OX415245.1	11	26.46	38.0
OX415246.1	MT	0.2	43.0
OX415247.1	Pltd	0.15	37.0

The estimated Quality Value (QV) of the final assembly is 71.9 with *k*-mer completeness of 100.0%, and the assembly has a BUSCO v5.3.2 completeness of 93.8% (single = 89.3%, duplicated = 4.5%), using the eudicots_odb10 reference set ( *n* = 2,326).

Metadata for specimens, barcode results, spectra estimates, sequencing runs, contaminants and pre-curation assembly statistics are given at https://links.tol.sanger.ac.uk/species/29803.

## Methods

### Sample acquisition, genome size estimation and nucleic acid extraction

A specimen of *Sherardia arvensis* (specimen ID KDTOL10195, ToLID daSheArve1) was picked by hand from the edge of a pavement at Teddington Lock, Surrey, UK (latitude 51.43, longitude –0.33) on 10th May 2021. The specimen was collected and identified by Maarten J. M. Christenhusz (Royal Botanic Gardens Kew) and preserved by freezing at –80°C.

The genome size was estimated by flow cytometry using the fluorochrome propidium iodide and following the ‘one-step’ method as outlined in Pellicer *et al.* (2021). Specifically for this species, CyStain™ PI OxProtect Staining Buffer (cat. No. 05-5027; Sysmex UK Ltd.) was used for isolation of nuclei. The internal calibration standard was *Petroselinum crispum* ‘Champion Moss Curled’ with an assumed 1C-value of 2,200 Mb ( Obermayer *et al.*, 2002).

The workflow for high molecular weight (HMW) DNA extraction at the Wellcome Sanger Institute (WSI) includes a sequence of core procedures: sample preparation; sample homogenisation, DNA extraction, fragmentation, and clean-up. In sample preparation, the daSheArve1 sample was weighed and dissected on dry ice ( Jay *et al.*, 2023). For sample homogenisation, leaf tissue was cryogenically disrupted using the Covaris cryoPREP ^®^ Automated Dry Pulverizer ( Narváez-Gómez *et al.*, 2023). HMW DNA was extracted using the Automated Plant MagAttract v2 protocol ( Todorovic *et al.*, 2023a). The DNA was sheared into an average fragment size of 12–20 kb in a Megaruptor 3 system with speed setting 30 ( Todorovic *et al.*, 2023b). Sheared DNA was purified by solid-phase reversible immobilisation ( Strickland *et al.*, 2023): in brief, the method employs a 1.8X ratio of AMPure PB beads to sample to eliminate shorter fragments and concentrate the DNA. The concentration of the sheared and purified DNA was assessed using a Nanodrop spectrophotometer and Qubit Fluorometer and Qubit dsDNA High Sensitivity Assay kit. Fragment size distribution was evaluated by running the sample on the FemtoPulse system.

RNA was extracted from leaf tissue of daSheArve1 in the Tree of Life Laboratory at the WSI using the RNA Extraction: Automated MagMax™ *mir*Vana protocol ( do Amaral *et al.*, 2023). The RNA concentration was assessed using a Nanodrop spectrophotometer and a Qubit Fluorometer using the Qubit RNA Broad-Range Assay kit. Analysis of the integrity of the RNA was done using the Agilent RNA 6000 Pico Kit and Eukaryotic Total RNA assay.

Protocols developed by the WSI Tree of Life core laboratory are publicly available on protocols.io ( Denton *et al.*, 2023).

### Sequencing

Pacific Biosciences HiFi circular consensus DNA sequencing libraries were constructed according to the manufacturers’ instructions. Poly(A) RNA-Seq libraries were constructed using the NEB Ultra II RNA Library Prep kit. DNA and RNA sequencing was performed by the Scientific Operations core at the WSI on Pacific Biosciences SEQUEL II (HiFi) and Illumina NovaSeq 6000 (RNA-Seq) instruments. Hi-C data were also generated from leaf tissue of daSheArve1 using the Arima2 kit and sequenced on the Illumina NovaSeq 6000 instrument.

### Genome assembly, curation and evaluation

Assembly was carried out with Hifiasm ( Cheng *et al.*, 2021) and haplotypic duplication was identified and removed with purge_dups ( Guan *et al.*, 2020). The assembly was then scaffolded with Hi-C data ( Rao *et al.*, 2014) using YaHS ( Zhou *et al.*, 2023). The assembly was checked for contamination and corrected using the gEVAL system ( Chow *et al.*, 2016) as described previously ( Howe *et al.*, 2021). Manual curation was performed using gEVAL, HiGlass ( Kerpedjiev *et al.*, 2018) and PretextView ( Harry, 2022). The organelle genomes were assembled using MitoHiFi ( Uliano-Silva *et al.*, 2023) and OATK ( Zhou, 2023).

A Hi-C map for the final assembly was produced using bwa-mem2 ( Vasimuddin *et al.*, 2019) in the Cooler file format ( Abdennur & Mirny, 2020). To assess the assembly metrics, the *k*-mer completeness and QV consensus quality values were calculated in Merqury ( Rhie *et al.*, 2020). This work was done using Nextflow ( Di Tommaso *et al.*, 2017) DSL2 pipelines “sanger-tol/readmapping” ( Surana *et al.*, 2023a) and “sanger-tol/genomenote” ( Surana *et al.*, 2023b). The genome was analysed within the BlobToolKit environment ( Challis *et al.*, 2020) and BUSCO scores ( Manni *et al.*, 2021; Simão *et al.*, 2015) were calculated.

Table 3 contains a list of relevant software tool versions and sources.

**Table 3.** Software tools: versions and sources.

Software tool	Version	Source
BlobToolKit	4.2.1	https://github.com/blobtoolkit/blobtoolkit
BUSCO	5.3.2	https://gitlab.com/ezlab/busco
gEVAL	N/A	https://geval.org.uk/
Hifiasm	0.16.1-r375	https://github.com/chhylp123/hifiasm
HiGlass	1.11.6	https://github.com/higlass/higlass
MBG	-	https://github.com/maickrau/MBG
Merqury	MerquryFK	https://github.com/thegenemyers/MERQURY.FK
MitoHiFi	2	https://github.com/marcelauliano/MitoHiFi
PretextView	0.2	https://github.com/wtsi-hpag/PretextView
purge_dups	1.2.3	https://github.com/dfguan/purge_dups
sanger-tol/genomenote	v1.0	https://github.com/sanger-tol/genomenote
sanger-tol/readmapping	1.1.0	https://github.com/sanger-tol/readmapping/tree/1.1.0
YaHS	yahs-1.1.91eebc2	https://github.com/c-zhou/yahs

### Wellcome Sanger Institute – Legal and Governance

The materials that have contributed to this genome note have been supplied by a Darwin Tree of Life Partner. The submission of materials by a Darwin Tree of Life Partner is subject to the **‘Darwin Tree of Life Project Sampling Code of Practice’**, which can be found in full on the Darwin Tree of Life website here. By agreeing with and signing up to the Sampling Code of Practice, the Darwin Tree of Life Partner agrees they will meet the legal and ethical requirements and standards set out within this document in respect of all samples acquired for, and supplied to, the Darwin Tree of Life Project. 

Further, the Wellcome Sanger Institute employs a process whereby due diligence is carried out proportionate to the nature of the materials themselves, and the circumstances under which they have been/are to be collected and provided for use. The purpose of this is to address and mitigate any potential legal and/or ethical implications of receipt and use of the materials as part of the research project, and to ensure that in doing so we align with best practice wherever possible. The overarching areas of consideration are:

• Ethical review of provenance and sourcing of the material

• Legality of collection, transfer and use (national and international) 

Each transfer of samples is further undertaken according to a Research Collaboration Agreement or Material Transfer Agreement entered into by the Darwin Tree of Life Partner, Genome Research Limited (operating as the Wellcome Sanger Institute), and in some circumstances other Darwin Tree of Life collaborators.

## Data Availability

European Nucleotide Archive: *Sherardia arvensis*. Accession number PRJEB50877; https://identifiers.org/ena.embl/PRJEB50877 ( Wellcome Sanger Institute, 2023). The genome sequence is released openly for reuse. The *Sherardia arvensis* genome sequencing initiative is part of the Darwin Tree of Life (DToL) project. All raw sequence data and the assembly have been deposited in INSDC databases. The genome will be annotated using available RNA-Seq data and presented through the Ensembl pipeline at the European Bioinformatics Institute. Raw data and assembly accession identifiers are reported in Table 1.

## Author information

Members of the Royal Botanic Gardens Kew Genome Acquisition Lab are listed here: https://doi.org/10.5281/zenodo.4786680.

Members of the Plant Genome Sizing collective are listed here: https://doi.org/10.5281/zenodo.7994306.

Members of the Darwin Tree of Life Barcoding collective are listed here: https://doi.org/10.5281/zenodo.4893703.

Members of the Wellcome Sanger Institute Tree of Life Management, Samples and Laboratory team are listed here: https://doi.org/10.5281/zenodo.10066175.

Members of Wellcome Sanger Institute Scientific Operations: Sequencing Operations are listed here: https://doi.org/10.5281/zenodo.10043364.

Members of the Wellcome Sanger Institute Tree of Life Core Informatics team are listed here: https://doi.org/10.5281/zenodo.10066637.

Members of the Tree of Life Core Informatics collective are listed here: https://doi.org/10.5281/zenodo.5013541.

Members of the Darwin Tree of Life Consortium are listed here: https://doi.org/10.5281/zenodo.4783558.
