# Supplementary material for: The genome sequence of field madder, Sherardia arvensis L., 1753 (Rubiaceae)
Source: Wellcome Open Res. 2024 Mar 1;9:126. [Version 1] doi: 10.12688/wellcomeopenres.21027.1 (PMC11320180; doi:10.12688/wellcomeopenres.21027.1)
Supplement: Peer review history

# Reviewer report: Reviewer response for version 1

**DOI:** 10.21956/wellcomeopenres.23263.r90556

**Published:** 2024-08-12

**Authors:** C. Robin Buell

**Affiliations:** 1 University of Georgia, Athens, GA, USA

This manuscript describes collection, sequence, assembly and quality assessment of the genome of field madder, *Sherardia arvensis* as part of the Darwin Tree of Life project.

 The authors used contemporary methods for the sequencing and assembly and provided detailed metrics of all aspects of their work along with open source, clear protocols.

 The genome has not been annotated and will be submitted to the ENsembl team for annotation; it would be good to link the genome sequence and this publication to the annotation once it is generated.

Are sufficient details of methods and materials provided to allow replication by others?

Yes

Is the rationale for creating the dataset(s) clearly described?

Yes

Are the datasets clearly presented in a useable and accessible format?

Yes

Are the protocols appropriate and is the work technically sound?

Yes

Reviewer Expertise:

Plant genome biology

I confirm that I have read this submission and believe that I have an appropriate level of expertise to confirm that it is of an acceptable scientific standard.

# Reviewer report: Reviewer response for version 1

**DOI:** 10.21956/wellcomeopenres.23263.r90562

**Published:** 2024-08-05

**Authors:** Rose A. Marks

**Affiliations:** 1 Michigan State University, East Lansing, Michigan, USA

1. **Rationale for Creating the Dataset:**
  - The paper clearly describes the rationale for creating the dataset. It provides comprehensive background information on *Sherardia arvensis*, highlighting its ecological significance and historical use. The rationale for sequencing the genome to understand the spread of native populations is well-articulated.
2. **Protocols and Technical Soundness:**
  - The protocols used in the study are appropriate and align with current best practices. The authors employed a robust combination of HiFi long reads, Hi-C data, and RNA-Seq. The technical methods are sound and well-executed.
3. **Details of Methods and Materials:**
  - Sufficient details are provided for methods and materials. The paper includes comprehensive descriptions of sample collection, DNA/RNA extraction, sequencing, and assembly processes. References to protocols and software tools are included.
4. **Dataset Presentation and Accessibility:**
  - The datasets are clearly presented in a usable and accessible format. The assembly metrics, scaffold information, and interactive visualizations are provided, along with links to raw data and assemblies deposited in public databases.

Overall, the paper is well-written, concise, and provides a high-quality genome assembly. The methods follow current best practices, and the detailed presentation of data and protocols helps promote reproducibility and accessibility.

Are sufficient details of methods and materials provided to allow replication by others?

Yes

Is the rationale for creating the dataset(s) clearly described?

Yes

Are the datasets clearly presented in a useable and accessible format?

Yes

Are the protocols appropriate and is the work technically sound?

Yes

Reviewer Expertise:

Molecular ecology, genomics, plant science, ecology, desiccation tolerance

I confirm that I have read this submission and believe that I have an appropriate level of expertise to confirm that it is of an acceptable scientific standard.
